# Supplementary material for: Dendritic cell-elicited B-cell activation fosters immune privilege via IL-10 signals in hepatocellular carcinoma
Source: Nat Commun. 2016 Nov 17;7:13453. doi: 10.1038/ncomms13453 (PMC5118541; doi:10.1038/ncomms13453)
Supplement: Supplementary Information — Supplementary Figures 1-5 and Supplementary Tables 1-5. [file ncomms13453-s1.pdf]

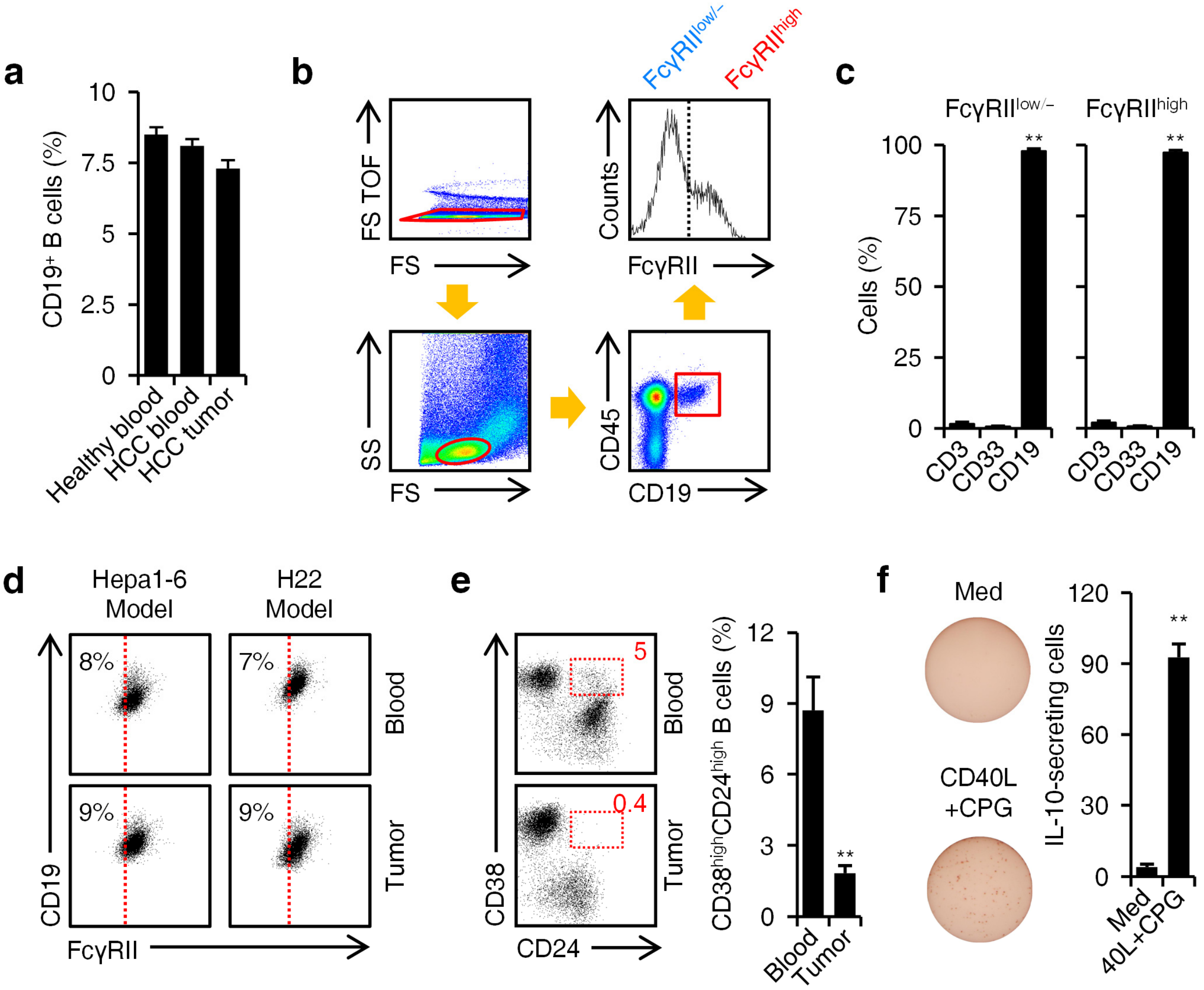

Supplementary Fig. 1: Proportion, subset composition, gating strategy of B cells from blood and HCC tumor samples.

(**a**) FACS analysis of $CD19^+$ B cells to $CD45^+$ mononuclear cells in healthy blood (n = 10) and paired blood and HCC tumor (n = 25) samples. (**b**) Gating strategy for FACS analysis. Mononuclear cells from blood or HCC tissue were first gated for singlets (FS-TOF vs. FS) and lymphocytes (SS vs. FS), and thereafter, the $CD45^+CD19^+$ B lymphocytes were further divided into $Fc\gamma RII^{low/-}$ and $Fc\gamma RII^{high}$ subsets. (**c**) Expression of CD3, CD19, and CD33 in HCC tumor-derived $Fc\gamma RII^{low/-}$ and $Fc\gamma RII^{high}$ B cells was determined by FACS (n = 5). (**d**) Murine Hepa1-6 and H22 hepatoma cells (10^6) in 25 µl Matrigel (Corning) were injected under the hepatic capsule of 5–7-week-old female C57BL/6 mice and BABL/c mice, respectively, for 25 days. Thereafter, the expression of FcγRII on blood and tumor B cells from both models was determined by FACS (n = 5 for each). (**e**) FACS analysis of $CD24^{high}CD38^{high}$ B cell proportions in B cells from paired blood and HCC tumor (n = 10). (**f**) Sorted blood $CD38^{high}CD24^{high}$ B cells (n = 5) were left untreated or stimulated with 1 µg/ml CD40L plus 2 µg/ml CpG for 24 h. IL-10 production was determined by ELISpot. $^*P < 0.05$, $^{**}P < 0.01$ (Student's t test). Error bars, s.e.m.

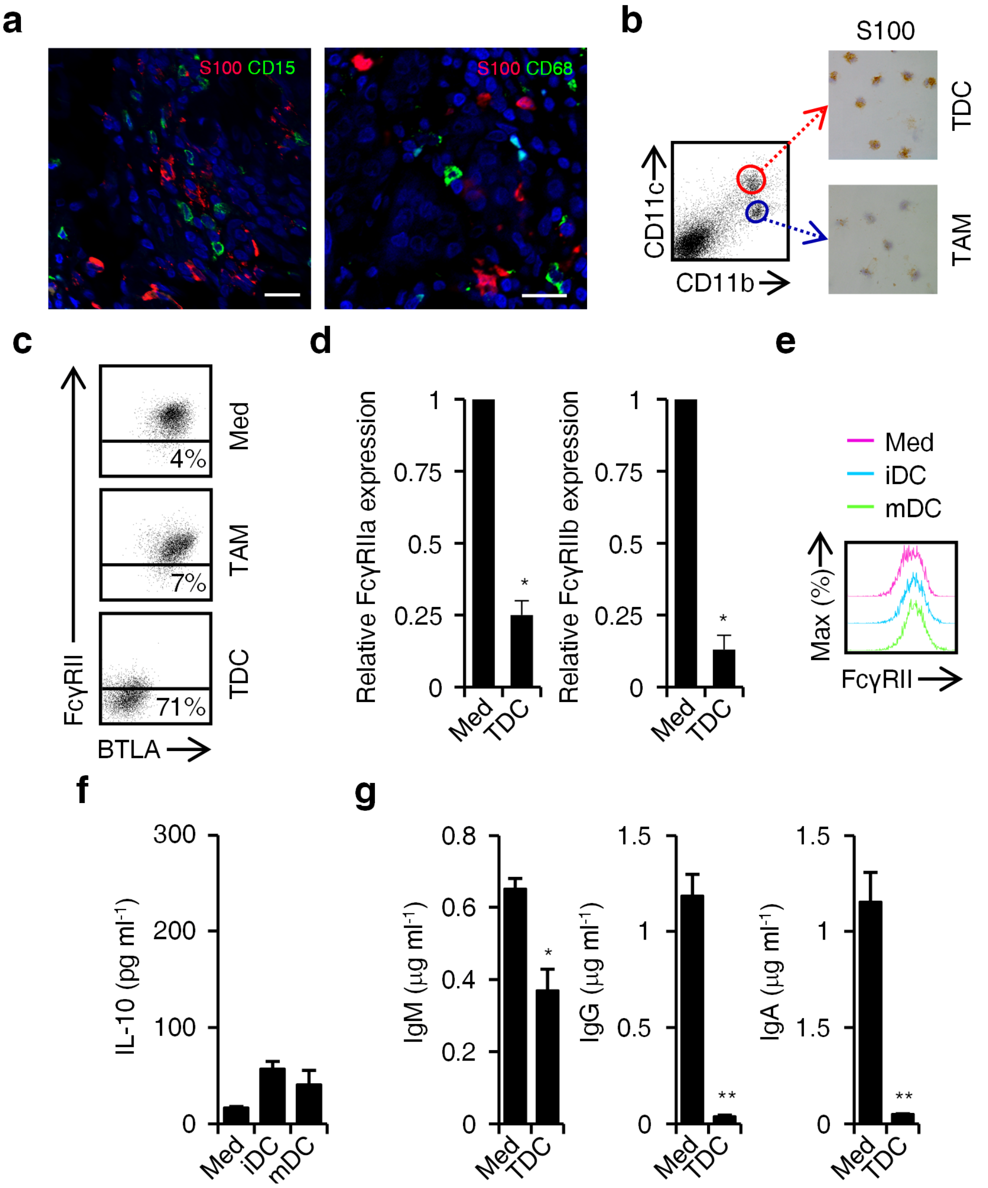

Supplementary Fig. 2: Effects of DCs on B cell activation, maturation, and IL-10 production. (**a**) Detection of S100 (red) and CD15 (green), or S100 (red) and CD68 (green) (n = 8) in peritumoral stroma by immunofluorescence. Scale bar, 50 µm. (**b**) Representative S100 staining in sorted TDCs and TAMs purified from HCC tumors (n = 4). (**c**) HCC tumor-derived TDCs, but not CD11c^{low}CD11b^{high} macrophages (TAMs), effectively induced FcγRII$^{low/–}$BTLA$^{low/–}$ B cells. (**d**) Blood B cells were left untreated or cultured with TDCs for 2 days. Thereafter, the B cells were sorted and the expression of FcγRIIa and FcγRIIb in these cells was determined by real-time PCR. (**e,f**) Effect of normal immature DCs (iDCs) and mature DCs (mDCs) on generation of IL-10-producing FcγRII$^{low/–}$ B cells. Peripheral CD19^{+} B cells were cultured in medium or with iDCs or mDCs for 24 hrs. Thereafter, B cells were purified by CD19^{+} beads and then cultured for additional 24 hr in medium alone. Expression of FcγRII in B cells was determined by FACS. Concentration of IL-10 in culture supernatant was determined by ELISA. (**g**) B cells left untreated (Med) or cultured with TDCs for 2 days were stimulated with CD40L plus IL-21 for 6 days. IgM, IgG and IgA production were determined by ELISA. Results (**c-g**) represent three independent experiments (n = 5). *P < 0.05, **P < 0.01 (Student's t test). Error bars, s.e.m.

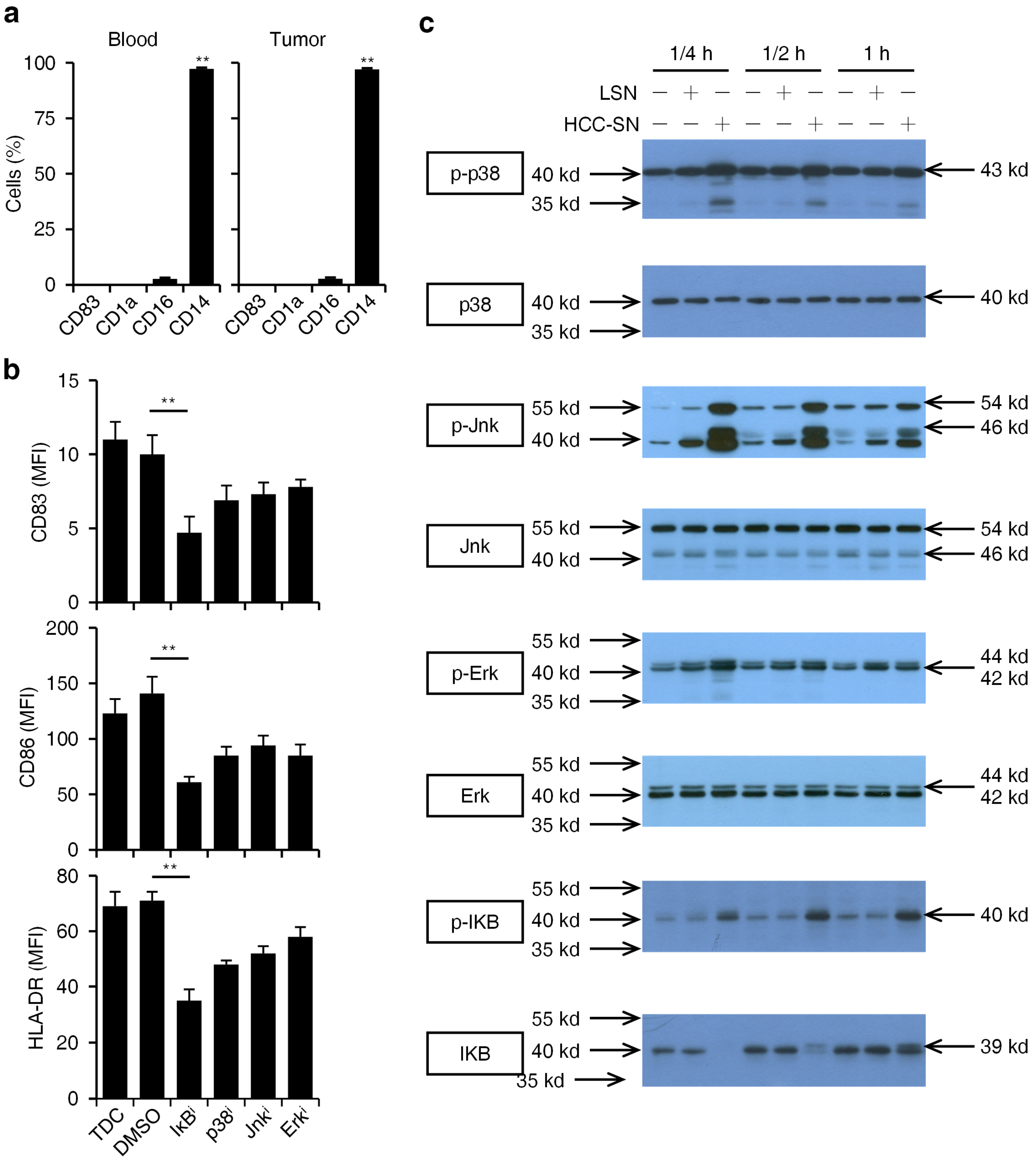

Supplementary Fig. 3: Effects of HCC-SN on DC maturation.

(**a**) Expression of CD1a, CD14, CD16, and CD83 in HCC blood- and tumor-derived CD14^{+} monocytes was determined by FACS (n = 5). (**b**) Suppression of IκBα, but not Erk1/2, p38, or Jnk, in HCC-SN-pretreated monocytes attenuated HCC-SN-mediated DC semimaturation. Expression of CD83, CD86, and HLA-DR was determined by FACS (n = 3). (**c**) Uncropped western blot images of Fig. 3g. *P < 0.05, **P < 0.01 (Student's t test). Error bars, s.e.m.

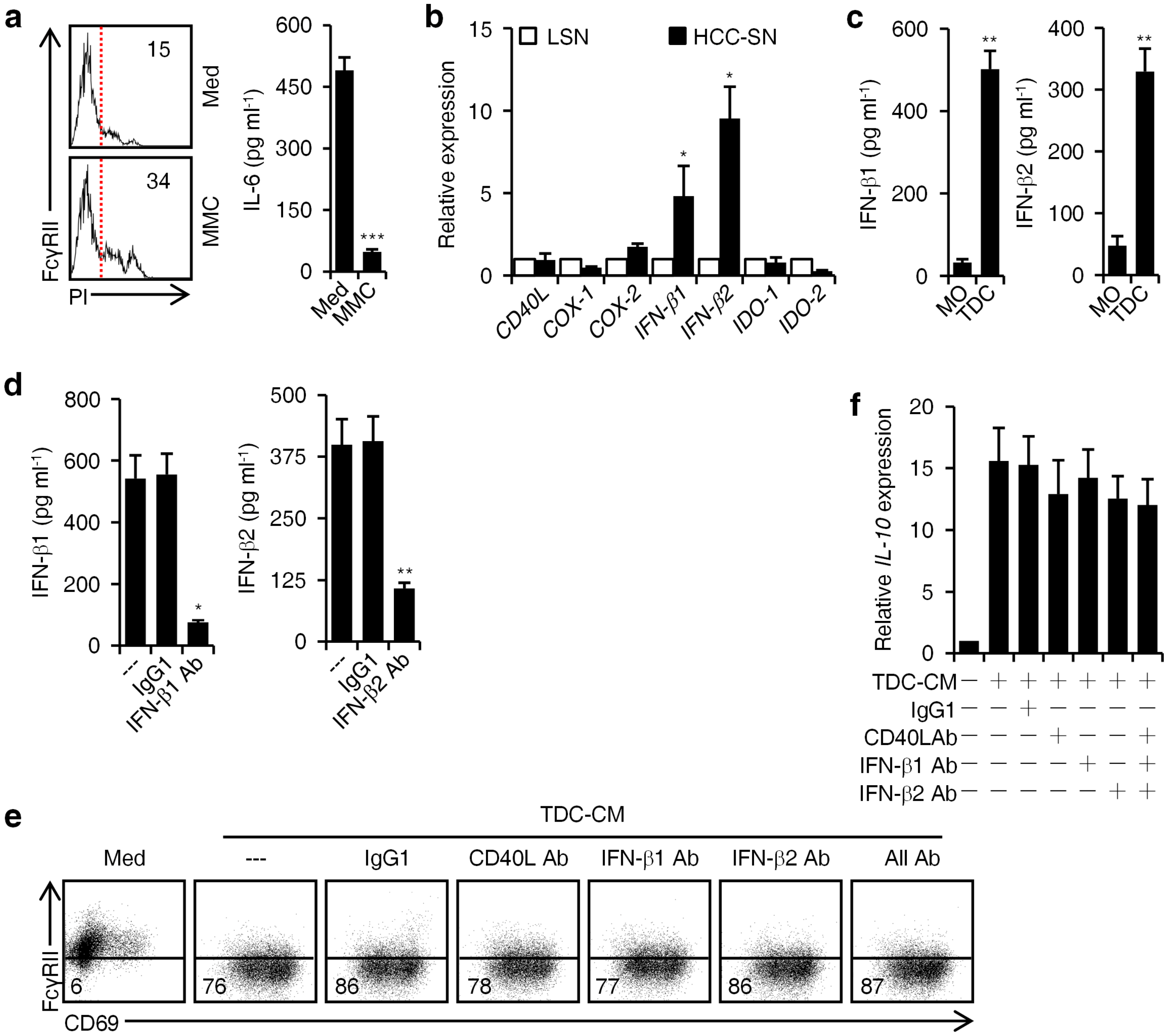

Supplementary Fig. 4: Effects of IFN-β1 and IFN-β2 on TDC-CM-elicited IL-10-producing FcγRII$^{low/-}$ B cell generation.

(**a**) Effect of mitomycin C (10 μg ml^{-1}) on TDC survival and cytokine production. (**b**) Real-time PCR analysis of immune regulatory molecule expression in DCs generated from blood monocytes that were pretreated for 1 hr with HCC-SN or LSN. (**c**) Detection of 24-hr IFN-β1 and IFN-β2 production in TDCs compared with paired blood monocytes using ELISA (n = 5). (**d-f**) Blocking IFN-β1, IFN-β2, and/or CD40L did not affect TDC-CM-elicited IL-10-producing FcγRII$^{low/-}$ B cell generation. Peripheral CD19^{+} B cells were cultured in medium or TDC-CM for 24 hrs. Thereafter, B cells were washed and then cultured for additional 24 hr in medium alone. The blocking effects of IFN-β1 and IFN-β2 in TDC-CM by specific mAbs were determined by ELISA (**d**). Expression of FcγRII in B cells was determined by FACS (**e**). Real-time PCR analysis of IL-10 expression in B cells (**f**). Results shown represent three independent experiments (n = 4). *P < 0.05, **P < 0.01 (Student's t test). Error bars, s.e.m.

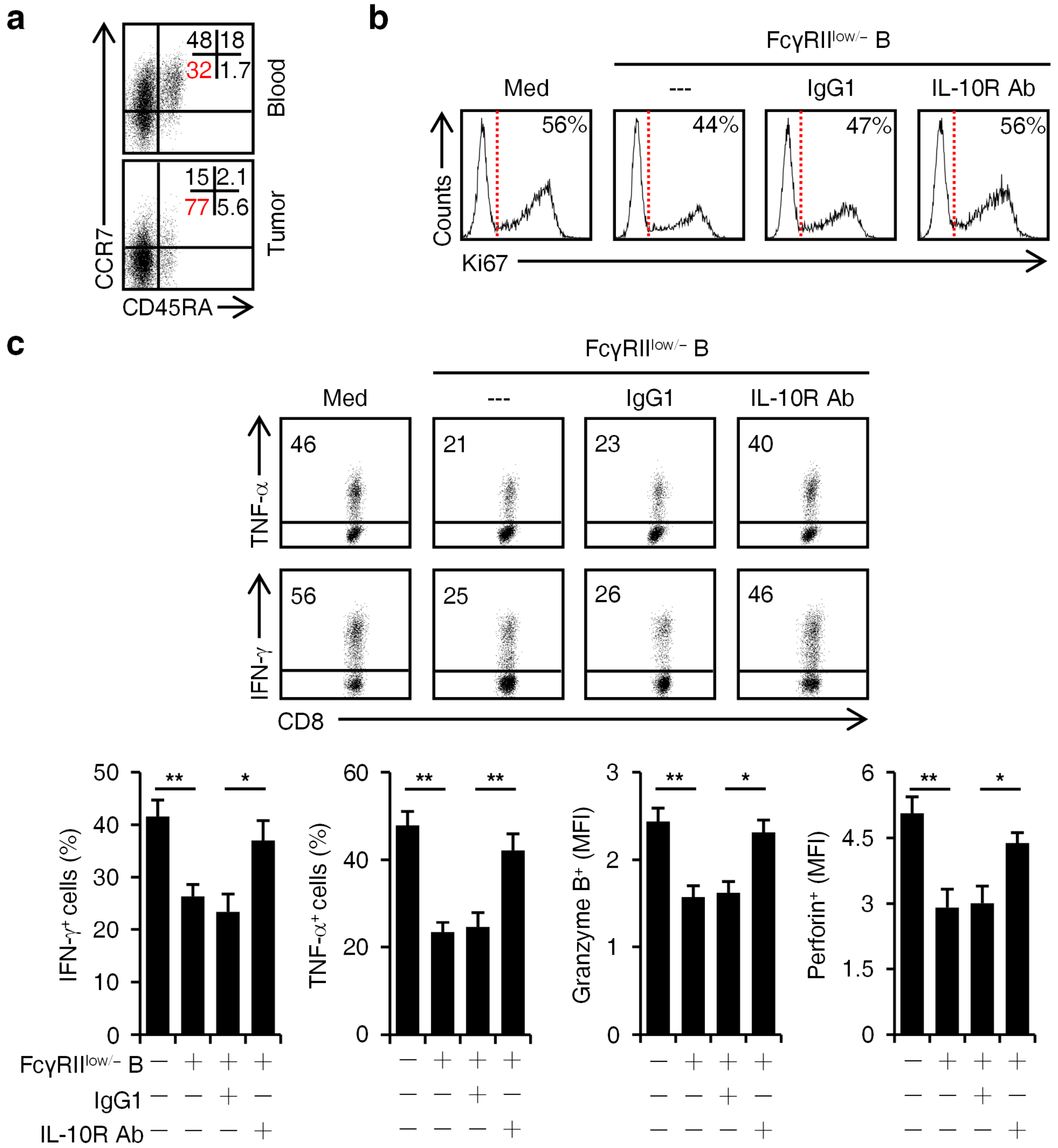

Supplementary Figure 5: Effects of tumor-derived or TDC-induced FcγRII$^{low/-}$ B cells on cytotoxic T cell immunity.

(**a**) Expression of CD45RA and CCR7 on CD8$^+$ T cells from paired blood and HCC tumor was determined by FACS (n = 6). (**b**) HCC tumor-derived CD8$^+$ T cells were left untreated or cultured with sorted tumor FcγRII$^{low/-}$ B cells in the presence or absence of an anti-IL10R or a control Ab. FACS analysis of Ki67 expression in CD8$^+$ T cells is shown. Results represent three independent experiments (n = 5). (**c**) CD8$^+$ T cells were left untreated or cultured with autologous HCC-SN/DC-induced FcγRII$^{low/-}$ B cells in the presence or absence of an anti-IL10R or a control Ab. Expression of proinflammatory TNF-α and IFN-γ and cytotoxic granzyme B and perforin in CD8$^+$ T cells activated by polyclonal stimulations was detected by FACS. Results represent three independent experiments (n = 3). $^*P < 0.05$, $^{**}P < 0.01$ and $^{***}P < 0.001$ (Student's t test). Error bars, s.e.m.

Supplementary Table 1. Clinical characteristics of 160 HCC Patients

Patient characteristics	Cohort 1	Cohort 2
No. of patients	25	135
Age, years (median, range)	53, 32-72	48, 24-75
Sex (male/female)	22/3	124/11
HbsAg (negative/positive)	8/17	14/121
Cirrhosis (absent/present)	10/15	20/115
ALT, U/L (median, range)	51.5, 24.3-484.2	41, 8-932
AFP, ng/ml (≤25/＞25)	16/9	44/91
Tumor size, cm (≤5/＞5)	11/14	62/73
Tumor multiplicity (solitary/multiple)	20/5	113/22
Vascular invasion (absent/present)	21/4	130/5
Intrahepatic metastasis (no/yes)	22/3	119/16
TNM stage (I+II/III+IV)	14/11	104/31
Tumor differentiation (I+II/III+IV)	7/18	110/25
Fibrous capsule (absent/present)	10/15	29/106
Nontumoral $S100^{+}$ cells density (median, range)	N/A	0.5, 0-34
Peritumoral stromal $S100^{+}$ cells (median, range)	N/A	20.5, 0-72.5
Intratumoral $S100^{+}$ cells (median, range)	N/A	0.2, 0-18

Supplementary Table 2: Univariate and multivariate analyses of factors associated with survival

Variables				Univariate	Multivariate		
				**P value**	**HR**	**95%CI of HR**	**P value**
Age, years	>48	vs.	≤48	**0.036**	1.521	0.859-2.693	0.151
Gender	female	vs.	male	0.405			
HBsAg	positive	vs.	negative	0.380			
Cirrhosis	high	vs.	absent	0.829			
ALT, U/L	>41	vs.	≤41	0.997			
AFP, ng/ml	>25	vs.	≤25	0.121			
Tumor size, cm	>5	vs.	≤5	**0.003**	1.707	0.902-3.232	0.100
Tumor multiplicity	multiple	vs.	solitary	**<0.001**	1.331	0.440-4.022	0.613
Vascular invasion	present	vs.	absent	0.097			
Intrahepatic metastasis	yes	vs.	no	**<0.001**	3.331	1.372-8.089	**0.008**
TNM stage	Ⅲ+Ⅳ	vs.	Ⅰ+Ⅱ	**0.006**	0.920	0.352-2.404	0.864
Tumor differentiation	Ⅲ+Ⅳ	vs.	Ⅰ+Ⅱ	0.147			
Fibrous capsule	present	vs.	absent	0.451			
Nontumoral $S100^+$ cells	>0.5	vs.	≤0.5	0.074			
Peritumoral stromal $S100^+$ cells	>20.5	vs.	≤20.5	**0.008**	1.973	1.059-3.675	**0.032**
Intratumoral $S100^+$ cells	>0	vs.	≤0	0.86			

Supplementary Table 3: Fluorochrome-conjugated antibodies used in flow cytometry

Species	Source	Antigen	Fluorochrome	Clone	Supplier
Human	Mouse	BTLA	PE	J168-540	BD Pharmingen
Human	Mouse	CCR1	Alexa Fluor 647	53504	BD Pharmingen
Human	Mouse	CCR2	Alexa Fluor 647	48607	BD Pharmingen
Human	Mouse	CCR6	PE	11A9	BD Pharmingen
Human	Rat	CCR7	PE	3D12	BD Pharmingen
Human	Armenian hamster	CCR10	PE	6588-5	Biolegend
Human	Mouse	CD11b	Alexa Fluor 700	CBRM1/5	eBioscience
Human	Mouse	CD11c	PE	3.9	eBioscience
Human	Mouse	CD14	FITC	61D3	eBioscience
Human	Mouse	CD15	Pacific Blue	MMA	eBioscience
Human	Mouse	CD16	FITC	3G8	BioLegend
Human	Mouse	CD19	PE-Cy7	J3-119	Beckman Coulter
Human	Mouse	CD19	BV421	HIB19	BioLegend
Human	Mouse	CD24	Alexa Fluor 780	eBioSN3	eBioscience
Human	Mouse	CD3	Alexa Fluor 700	OKT3	eBioscience
Human	Mouse	CD32	APC	6C4 (CD32)	eBioscience
Human	Mouse	CD32	PE-Cy7	6C4 (CD32)	eBioscience
Human	Mouse	CD33	APC	D3HL60.251	Beckman Coulter
Human	Mouse	CD38	FITC	HIT2	BD Pharmingen
Human	Mouse	CD45	Krome Orange	J.33	Beckman Coulter
Human	Mouse	CD45RA	Pacific Blue	HI100	BioLegend
Human	Mouse	CD45RO	PE	UCHL1	BD Pharmingen
Human	Mouse	CD62L	PE	DREG-56	BD Pharmingen
Human	Mouse	CD69	PE	TP1.55.3	Beckman Coulter
Human	Mouse	CD8	FITC	B9.11	Beckman Coulter
Human	Mouse	CD83	FITC	HB15e	BioLegend
Human	Mouse	CD86	APC	2331 (FUN-1)	BD Pharmingen
Human	Mouse	CD95	PE-Cy5	DX2	eBioscience
Human	Rat	CXCR5	Alexa Fluor 647	RF8B2	BD Pharmingen
Human	Mouse	HLA-DR	Pacific Blue	Immu-357	Beckman Coulter
Human	Mouse	Granzyme B	PE	GB11	eBioscience
Human	Mouse	Perforin	APC	delta G9	eBioscience
Human	Rat	IFN-γ	Alexa Fluor 647	XMG1.2	BD Pharmingen
Human	Mouse	IFN-γ	PE	45.15	Beckman Coulter
Human	Mouse	TNF-α	PE	IPM2	Beckman Coulter
Human	Mouse	IgM	FITC	UHB	Beckman Coulter
Human	Goat	IgA	PE	N/A	SouthernBiotech
Human	Mouse	IgD	Alexa Fluor 700	IA6-2	BD Pharmingen
Human	Mouse	IgG	APC-H7	G18-145	BD Pharmingen
Human	Rat	IL-10R	APC	3F9	BioLegend
Human	Mouse	Ki67	PE	B56	BD Pharmingen

Supplementary Table 4: Primers for real-time PCR

Gene		Sequence
Human *CD95L*	Forward	GGT CCA TGC CTC TGG AAT GG
	Reverse	CAC ATC TGC CCA GTA GTG CA
Human *CD40L*	Forward	AAT TGC GGC ACA TGT CAT AA
	Reverse	GTT TCC CAT TTT CCA GGG TT
Human *COX-1*	Forward	TAC CAG GTG CTG GAT GGA GA
	Reverse	CCT TCA GCA GGT CAC ACA C
Human *COX-2*	Forward	GTT TTG ACA TGG GTG GGA AC
	Reverse	CCC TCA GAC AGC AA AGC CTA
Human *IFN-β1*	Forward	CGA CAC TGT TCG TGT TGT CA
	Reverse	GAA GCA CAA CAG GAG AGC AA
Human *IFN-β2*	Forward	TCA GCC CTG AGA AAG GAG ACA
	Reverse	GAT TTT CAC CAG GCA AGT CTC C
Human *IDO-1*	Forward	ACA GAC CAC AAG TCA CAG CG
	Reverse	TTT GCC AAG ACA CAG TCT GC
Human *IDO-2*	Forward	CTG GTC CTG AGC TTC CTC AC
	Reverse	CAG CAC CAA GTC TGA GTG GA
Human *IL-10*	Forward	CAA CCT GCC TAA CAT GCT TC
	Reverse	CCT TGA TGT CTG GGT CTT GG
Human *GAPDH*	Forward	GAG TCA ACG GAT TTG GTC GT
	Reverse	GAC AAG CTT CCC GTT CTC AG

Supplementary Table 5: Antibodies against human antigens used in immunoblotting, IHC, and IF

Antigen	Source	Clone	Supplier	Dilution	Application
Phospho-p38	Rabbit	D3F9	Cell Signaling Technology	1:1000	immunoblotting
p38	Rabbit	D13E1	Cell Signaling Technology	1:1000	immunoblotting
Phospho-Jnk	Rabbit	98F2	Cell Signaling Technology	1:1000	immunoblotting
Jnk	Rabbit	56G8	Cell Signaling Technology	1:1000	immunoblotting
Phospho-Erk	Rabbit	20G11	Cell Signaling Technology	1:1000	immunoblotting
Erk	Rabbit	137F5	Cell Signaling Technology	1:1000	immunoblotting
Phospho- IκBα	Rabbit	14D4	Cell Signaling Technology	1:500	immunoblotting
IκBα	Mouse	L35A5	Cell Signaling Technology	1:1000	immunoblotting
CD20	Rabbit	EP7	ZSBio	1:200	IHC/IF
S100	Mouse	15E2E2+4C4.9	ZSBio	1:200	IHC
CD68	Mouse	PG-M1	Dako	1:200	IHC
CD8	Mouse	Ab-3	Neomarkers	1:200	IHC
